# *Fgfr1 *signalling in the development of a sexually selected trait in vertebrates, the sword of swordtail fish

**DOI:** 10.1186/1471-213X-8-98

**Published:** 2008-10-09

**Authors:** Nils Offen, Nicola Blum, Axel Meyer, Gerrit Begemann

**Affiliations:** 1 Lehrstuhl für Zoologie und Evolutionsbiologie, Department of Biology, University of Konstanz, D-78457 Konstanz, Germany

## Abstract

**Background:**

One of Darwin's chosen examples for his idea of sexual selection through female choice was the "sword", a colourful extension of the caudal fin of male swordtails of the genus *Xiphophorus*. Platyfish, also members of the genus *Xiphophorus*, are thought to have arisen from within the swordtails, but have secondarily lost the ability to develop a sword. The sustained increase of testosterone during sexual maturation initiates sword development in male swordtails. Addition of testosterone also induces sword-like fin extensions in some platyfish species, suggesting that the genetic interactions required for sword development may be dormant, rather than lost, within platyfish. Despite considerable interest in the evolution of the sword from a behavioural or evolutionary point of view, little is known about the developmental changes that resulted in the gain and secondary loss of the sword. Up-regulation of *msxC *had been shown to characterize the development of both swords and the gonopodium, a modified anal fin that serves as an intromittent organ, and prompted investigations of the regulatory mechanisms that control *msxC *and sword growth.

**Results:**

By comparing both development and regeneration of caudal fins in swordtails and platyfish, we show that *fgfr1 *is strongly up-regulated in developing and regenerating sword and gonopodial rays. Characterization of the fin overgrowth mutant *brushtail *in a platyfish background confirmed that fin regeneration rates are correlated with the expression levels of *fgfr1 *and *msxC*. Moreover, *brushtail *re-awakens the dormant mechanisms of sword development in platyfish and activates *fgfr1/msxC*-signalling. Although both genes are co-expressed in scleroblasts, expression of *msxC *in the distal blastema may be independent of *fgfr1*. Known regulators of Fgf-signalling in teleost fins, *fgf20a *and *fgf24*, are transiently expressed only during regeneration and thus not likely to be required in developing swords.

**Conclusion:**

Our data suggest that Fgf-signalling is involved upstream of *msxC *in the development of the sword and gonopodium in male swordtails. Activation of a gene regulatory network that includes *fgfr1 *and *msxC *is positively correlated with fin ray growth rates and can be re-activated in platyfish to form small sword-like fin extensions. These findings point towards a disruption between the *fgfr1/msxC *network and its regulation by testosterone as a likely developmental cause for sword-loss in platyfish.

## Background

**Figure 1:**
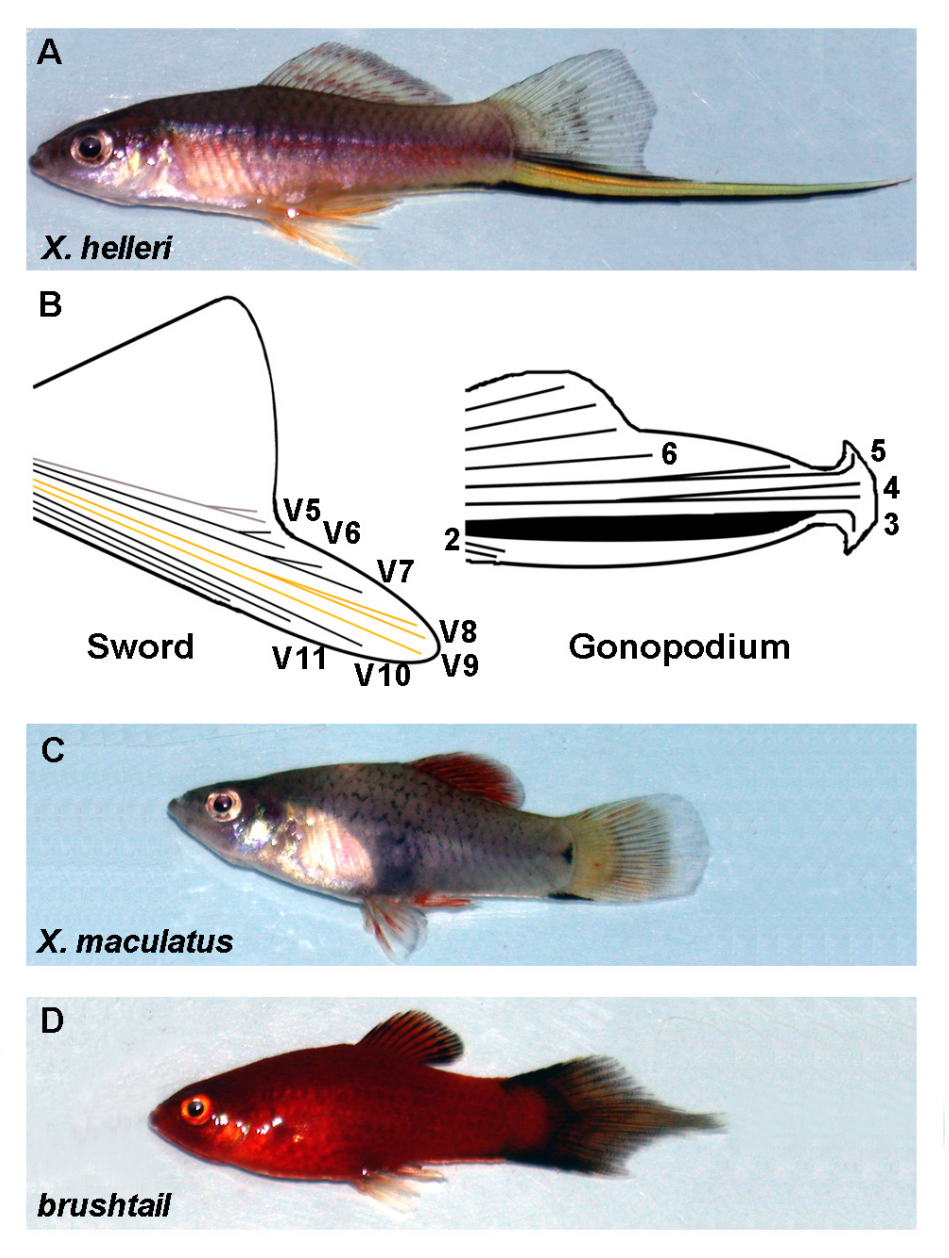
***Xiphophorus *species/strains used**. Adult morphologies of *X. helleri *(A) and *X. maculatus *(C) used in this study as representatives of the swordtail and platyfish lineages. Overview and nomenclature of adult fin rays (B) in the sword [21] of *X. helleri *and in the gonopodium [33] that is formed by swordtails and platyfish males. The caudal fin ray overgrowth mutant *brushtail *(D). Note that C and D show different strains of *X. maculatus *that exhibit dissimilar body colouration independent of the *brushtail *mutation.

Charles Darwin conceived not only the theory of natural selection, but also recognized that a theory of sexual selection is necessary to explain the presence of conspicuous traits in male animals that could not have arisen by natural selection [1]. A number of studies provided evidence that sexual selection increases taxonomic diversity, although it remains somewhat controversial if and how sexual selection alone can cause speciation (reviewed in [2,3]). The body of theory about sexual selection has been extended through several new insights that explain the evolution of sexually selected traits and mating behaviour. Fishes of the genus *Xiphophorus *are a popular model in which various aspects of sexual selection have been studied extensively (reviewed in [4]). The most prominent sexually selected trait in male swordtail fish of the genus is the sword, a conspicuously pigmented elongation of the ventral caudal fin. The sword consists of several components, i.e. a ventral fin elongation and a characteristic pigmentation pattern [5,6]. In the green swordtail *X. helleri*, it consists of centrally located yellow-orange or green coloured rays, that are flanked dorsally and ventrally by rays with strong melanisation (Figure 1A, B and [7]). Both length and colouration are important for mating success [7,8].

The evolutionary history of the sword is of particular interest. One scenario, supported by molecular phylogenies, suggests that all extant *Xiphophorus *species, swordtails and sword-less platyfish, descended from a common, sworded ancestor [5,9,10]. Moreover, short extensions of the ventral portion of the caudal fin are also phylogenetically widespread and, for example, are found in *Poecilia petenensis *[11]. Platyfish (Figure 1C), a common name that is used to describe several swordless species that belong to a monophyletic clade within the genus *Xiphophorus*, however, secondarily lost their sword during evolution, possibly because the costs in terms of natural selection were higher than the gain in terms of sexual selection. Nonetheless, females of some platy species in which this has been tested still prefer sworded males over the swordless males of their own species [12,13]. The preference for elongated caudal fins seems to be much older than the trait itself, since it is also present in at least one species of the sister genus *Priapella *[14]. Therefore, the sword is thought to have evolved in response to a pre-existing female bias, such as a general preference for the apparent size [8,12]. Due to this interesting evolutionary history, the sword presents a valuable model to study how evolution acts at the molecular level to generate or abolish a sexually selected trait. This objective has also driven research in other animals, e.g. the colour morphs in males of the livebearing fish *Poecilia parae *[15], the exaggerated hypercephaly in stalk-eyed flies [16], or the horns of dung beetles [17]. All of these are examples of model systems in which the basis of change in male exaggerated traits under sexual selection is amenable to genetic dissection.

One way to address this question in swordtails is to dissect the genetic pathways that might be involved in the development of the sword and to characterize these within a phylogenetic framework of the entire genus that involves swordtails and platyfish. So far, only little is known about the molecular basis of sword development. Hybridisation experiments between *X. helleri *and *X. cortezi *revealed that multiple genes control sword development, which were collectively termed "sword genes" ("Schwertgene"), i.e. genes or alleles that confer an ability to produce a sword in hybrids of platyfish and swordtails [18]. In addition, fin ray transplantation experiments have shown that sword rays are characterized by the possession of an organizing activity that induces neighbouring fin rays to contribute to the sword [19]. Sword-induction experiments with juvenile swordtails, treated with exogenous testosterone, revealed that testosterone is a sufficient and essential factor that induces sword development [20,21]. Exogenous testosterone also induces the development of the gonopodium (Figure 1B), a modified anal fin used as a copulation organ that is common to all fish in the family Poeciliidae (the livebearing toothcarps). This might suggest that androgen signalling regulates a molecular pathway that induces both sword and gonopodium development. Interestingly, some platy species develop a small ventral extension of the caudal fin through testosterone treatment [20-22], suggesting that the genetic machinery underlying sword development is still partly intact even in normally swordless platyfishes. Most likely, this machinery has never been lost completely, even though it might have been inactive for more than a million years [9,10].

Genes that regulate growth-dependent processes like fin regeneration are good candidates for genes involved in sword development. A candidate gene approach revealed *msxC *(*muscle segment homeobox* gene C), a gene known to act in fin regeneration, to be specifically up-regulated in developing swords and gonopodia [23]. By combining available genetic and phylogenetic data, it was hypothesized that genes and pathways that shape the evolutionarily older gonopodium have been partly adapted for sword development [23].

Other putative candidate genes for sword development are upstream regulators of *msxC*, such as components of the Fgf (Fibroblast growth factor) signalling pathway. Fgf signalling controls epithelial-mesenchymal interactions in the external genital anlagen of mammalian embryos [24]. Fibroblast growth factor receptor 1 (Fgfr1) appears to regulate *msxC *and *msxB *expression during caudal fin regeneration in zebrafish and is required for regenerative outgrowth of fin rays [25,26]. Furthermore, Fgf ligands such as those encoded by the *fgf24 *and *fgf20a *genes have been shown to play a role in caudal fin regeneration or pectoral fin development [27,28]. To test a putative role of Fgf-signalling in sword development we cloned the *fgf receptor 1 *and two *fgf *orthologs, *fgf24 *and *fgf20a*, from the swordtail *X. helleri *and analysed their expression pattern in developing swords and gonopodia as well as regenerating swords. From a developmental point of view, we asked whether regulation of *fgf *genes expression is associated with growth of the sword and gonopodium during development and sword regeneration. From an evolutionary standpoint, we were interested in evaluating whether potential differences in *fgf *gene expression between swordtails and platy species contribute to the understanding of the molecular changes that led to the loss of the sword during evolution. Furthermore, we analysed the expression of *fgfr1 *and *msxC *in regenerating caudal fins in the platyfish *X. maculatus *fin overgrowth mutant *brushtail*, where medial rays of the caudal fin continue to grow throughout the entire life of the animal (Figure 1D). We show that genes are regulated similarly in regenerating sword rays and elongated brush rays, although sword regeneration proceeds differently from regeneration in *brushtail*.

## Results

### Cloning and analysis of *fgf *genes

**Figure 2:**
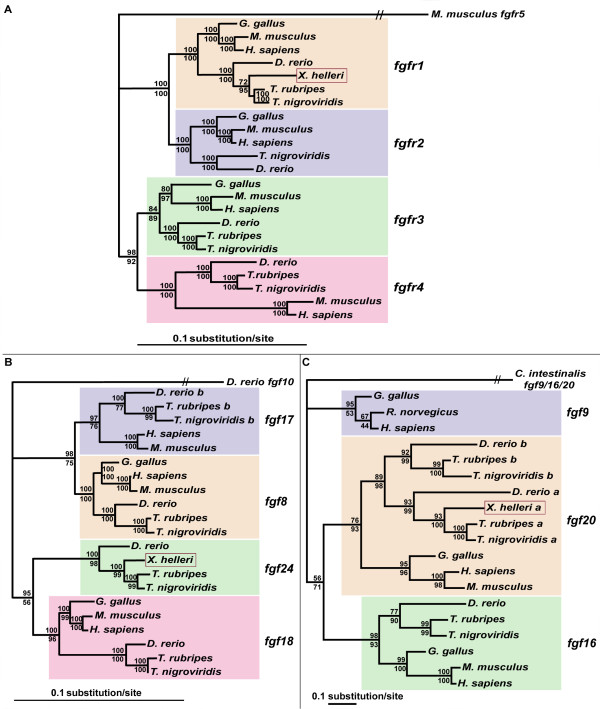
**Phylogeny of *fgf *genes**. Phylogenetic analysis of vertebrate *fgf receptors *(A), *fgf8/17/18/24 *(B) and *fgf9/16/20 *(C) families using PhyML (upper values) and Mr. Bayes (lower values). For analysis the coding regions of *fgf *genes cDNAs were used. The position of the *X. helleri *orthologs of *fgfr1*, *fgf24 *and *fgf20a *within the three phylogenies is highlighted (red box).

Since sword development in *X. helleri *requires the growth of caudal fin rays, genes that act to regulate growth in the regenerating zebrafish caudal fin appeared to be suitable candidate genes that may also be involved in sword development. Up-regulation of *fgfr1 *and *msx *gene expression has originally been observed in the blastema during zebrafish fin regeneration [25], and it had subsequently been shown that inhibition of Fgf signaling during ongoing fin regeneration prevents further outgrowth and down-regulates the established expression of blastemal *msx *genes [25,26,29,30]. *fgf24 *and *fgf20a *encode putative Fgfr1 ligands that are expressed or fulfill important functions in zebrafish fin regeneration [25,28]. To clone the *Xiphophorus *orthologs from the green swordtail, *Xiphophorus helleri*, we used a RT-PCR strategy and caudal fin blastemata as source for mRNA. The amplified fragment of the fibroblast growth factor receptor 1 (EU340805) covers 1248 bp of the protein's open reading frame, including parts of the IG Domain II, the complete IG Domain III and parts of the tyrosine kinase domain (see Figure A and B in Additional file 1), found in vertebrate Fgfr1 [31]. Phylogenetic reconstruction of the fgf receptor family, using coding sequence, confirmed that we cloned a partial sequence of the *X. helleri *Fgfr1 ortholog (Figure 2A).

The two cloned cDNA fragments of *fgf24 *include the complete coding sequence of *fgf24*, parts of the 5'UTR and the whole 3'UTR sequence. The 633 bp ORF (EU340806) of *X. helleri fgf24 *codes for a 210 amino acid protein with a heparin-binding growth factors/fibroblast growth factor (HBGF/FGF) family signature (see Figure A and B in Additional file 2). Phylogenetic analysis of the 633 bp cDNA sequence verified the sequence to be the *X. helleri fgf24 *ortholog (Figure 2B).

In addition we cloned two cDNA fragments of *fgf20a*, that together cover most of the coding and the complete 3'UTR sequence. The partial protein sequence, coded by 663 bp (EU340807), shows a conserved HBGF/FGF motif (see Figure A and B in Additional file 2). Interestingly, there is a QH-rich (aa 22–55) motif close the N-terminus of the sequence (see Figure A in Additional file 2). This motif could not be found in Fgf20a sequences of other vertebrate species. The phylogenetic analysis of the coding sequence confirmed it to be the *X. helleri fgf20a *ortholog (Figure 2C).

### *fgfr1 *and *msxC *are differently regulated in caudal fins of maturing swordails and platyfish

In order to test whether Fgf-signalling is involved in sword development of the green swordtail, *X. helleri *(Figure 1A), we treated 4–5 month old juvenile fish with 17-α-methyltestosterone to artificially induce this process. To allow both for the simultaneous generation of large numbers of experimental animals and for timed induction of sword development we prematurely induced swords in juvenile fish. Importantly, hormonally induced swords in immature juveniles do not show any sex-related morphological differences [18,21]. Even adult females develop a sword under testosterone treatment that is indistinguishable from the male sword both in length and pigmentation [18], therefore the sex of the individual should not bias the downstream analysis.

**Figure 3:**
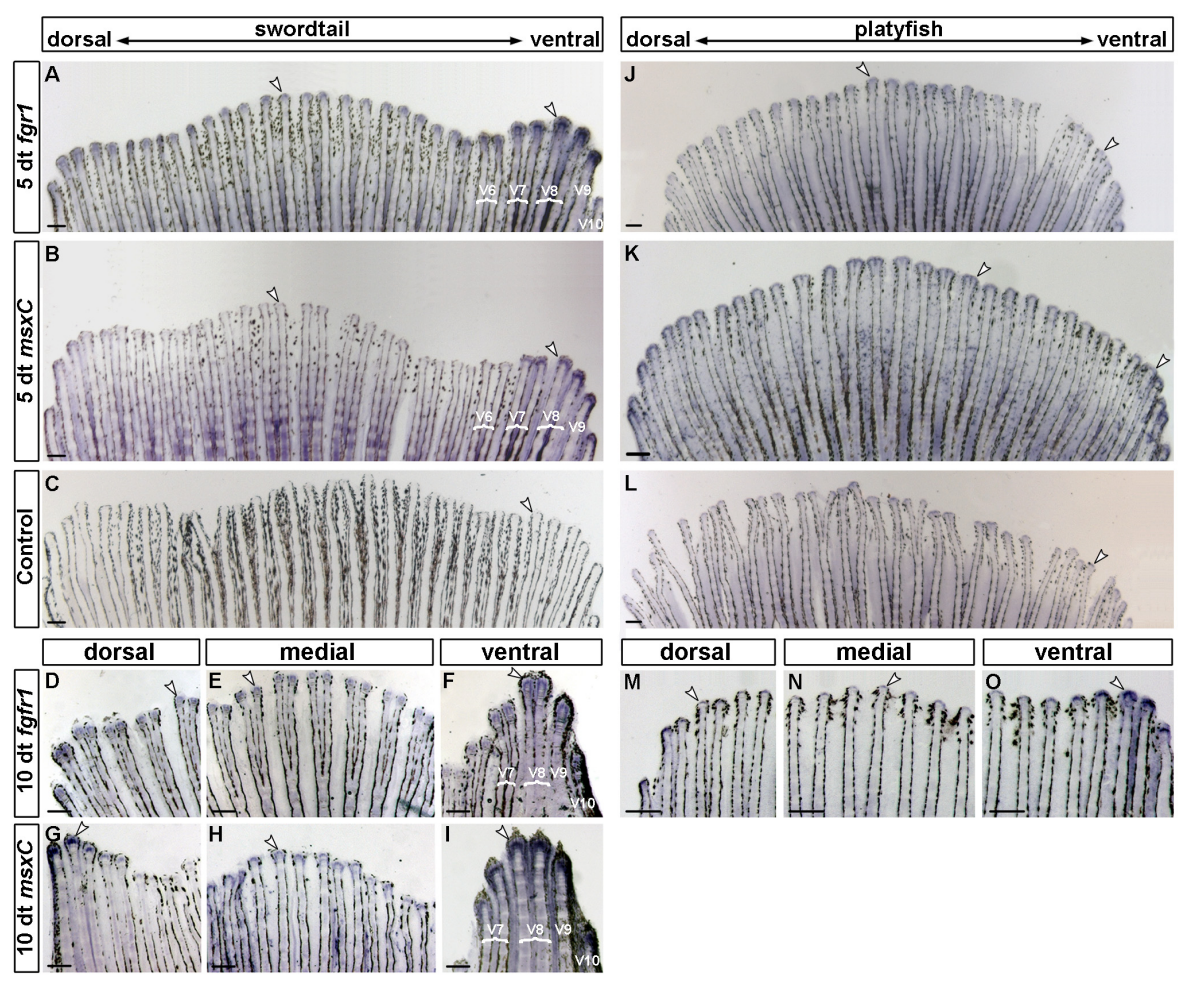
**Expression of *fgfr1 *and *msxC *in the developing sword**. *X. helleri fgfr1 is *up-regulated during sword development. When maturation is induced by exogenous testosterone, *fgfr1 *is up-regulated in the ventral-most caudal fin rays in developing swords at 5 and 10 days of treatment (dt)(A, F). Weaker expression of *fgfr1 *can also be detected in non-sword rays (A, D, E). *fgfr1 *is not up-regulated in untreated control fins (C). *fgfr1 *expression overlaps with *msxC*, which is also up-regulated in developing sword rays ([23], B and I). In later stages of treatment, up-regulation of both genes in the distal part of the dorsal-most rays is observed in some individuals (G), which may develop a small "upper sword" ([20]). Like *fgfr1, msxC *expression is also detected in non-sword rays (G, H). When maturation is induced by exogenous testosterone in the platyfish, *X. maculatus fgfr1 *and *msxC *are similarly expressed in all caudal fin rays after 5 dt (J and K). The expression levels are comparable to untreated fish (L). After 10 dt *fgfr1 *is more strongly expressed in the ventral-most fin-rays (O) compared to other rays (M, N), which may correspond to the formation of a small ventral swordlet [20,22]. White arrowheads indicate gene expression. (*X. helleri*: n = 10 for every stage and probe; *X. maculatus*: 5 dt: n = 5; 10 dt and controls: n = 3; scale bars: 200 μm)

In developing swords, *fgfr1 *expression was first observed after 4 days of hormone treatment (dt), when black pigmentation along the dorsal border of the sword also becomes visible (data not shown). After 5 dt, when the outgrowth of sword rays had started, *fgfr1 *was mainly up-regulated in the distal tip of the main ventral sword-forming fin rays V7–V10 (Figure 1B and [21]) compared to median or dorsal rays (Figure 3A). However, *fgfr1 *was expressed much more strongly in V7–V9 than in V10 (Figure 3A). A slight up-regulation of *fgfr1 *was also detected in ray V6 (Figure 3A). Importantly, this pattern is comparable to that of *msxC *a gene that is strongly up-regulated in developing swords (Figure 3B and [23]). Up-regulation of *fgfr1 *was not observed in control fins (Figure 3C). This overlap in expression pattern of *fgfr1 *and *msxC *persists during later stages of sword outgrowth (compare Figures 3D–F and 3G–I). These finding suggest that high levels of *fgfr1 *expression correlate with the development of ventral caudal fin rays into swords. Furthermore, the spatio-temporal overlap of both *fgfr1 *and *msxC *expression patterns indicate a likely interaction of these genes during sword development.

To test if changes in the regulation of *fgfr1 *and *msxC *are linked to the absence of the sword in platyfish, we assayed the expression of both genes in the caudal fin of the platyfish *X. maculatus *(Figure 1C) after 5 and 10 days of testosterone treatment. The expression of both genes in caudal fins at 5 dt differs clearly between *X. helleri *and *X. maculatus*. In *X. maculatus *both genes are uniformly expressed with no differences between sword and non-sword rays (Figures 3J and 3K). In addition, the expression patterns of both genes in testosterone treated fins are quite similar to those of control fins (Figure 3L). At 10 dt however, both genes are up-regulated in a subset of ventral fin rays (Figures 3M–O and not shown). This expression pattern is likely to mark the fin rays that will form a small caudal extension under high exogenous levels of testosterone [20,22]. Based on the expression data, we conclude that loss of sword ray specific regulation of *fgfr1 *or *msxC *could have been involved in the secondary loss of this trait in platyfish.

### *fgfr1 *and *msxC *regulation in maturing anal fins is conserved between swordtails and platyfish

Males of both platyfish and swordtails as juveniles possess typical anal fins, that during sexual maturation transform into a gonopodium, an intromittent organ for internal fertilisation [32]. Despite some differences in gonopodium morphology between species, all gonopodia are formed by anal fin rays 3–5 that develop into a structure that can deliver sperm into the females genital tract as well as scrape out sperm of other males through hooks that are formed by modification of fin ray elements (Figure 1B and [32,33]). We expected that gonopodia of both swordtails and platyfish show a similar spatio-temporal pattern of *fgfr1* and *msxC *expression. Because it was impractical to identify sufficient numbers of normally developing male juvenile fish at the desired stages, we analysed the expression patterns of both genes in artificially induced gonopodia of the swordtail *X. helleri *and the platyfish *X. maculatus*.

**Figure 4:**
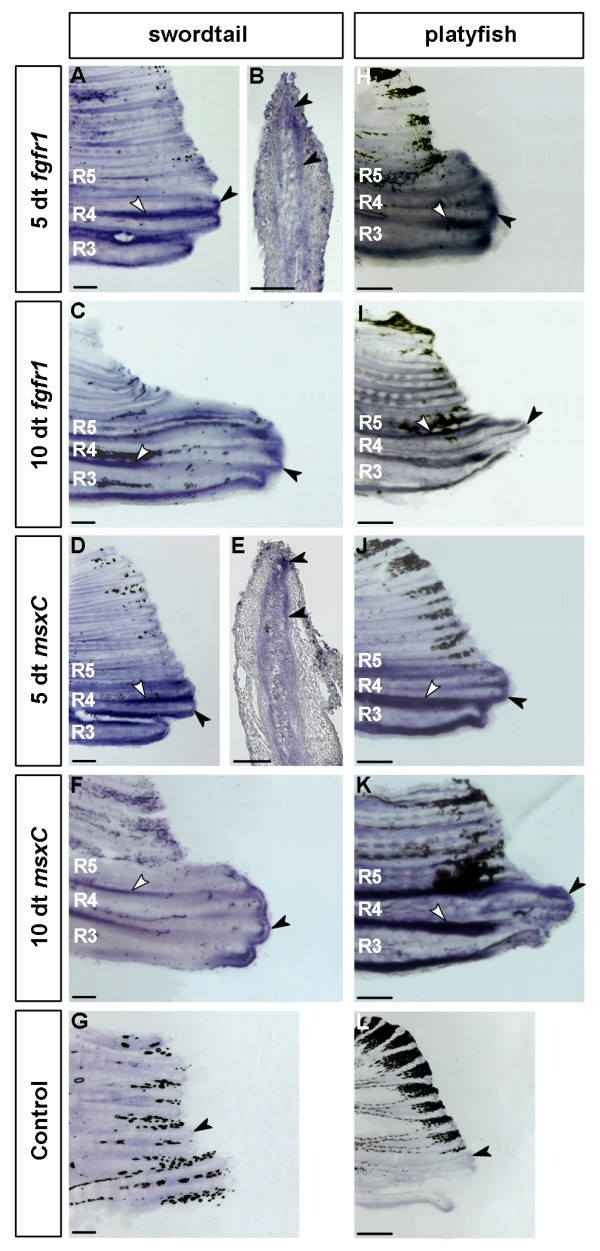
**Expression of *fgfr1 *and *msxC *in the developing gonopodia of *X. helleri *and *X. maculatus***. *fgfr1 *and *msxC *are both expressed in developing gonopodia of *X. helleri *and *X. maculatus*. In *X. helleri fgfr1 *is up-regulated at 5 days (A) and 10 days (C) of treatment in mesenchymal cells (B) of the main gonopodium-forming rays 3–5 compared to control fins (G). In addition *fgfr1 *is strongly expressed in the interray tissue of those rays (A, C). As in developing swords, *fgfr1 *expression overlaps with *msxC *expression (D-F). In early stages of gonopodium development (5 dt) of the platyfish *X. maculatus*, the expression patterns of *fgfr1 *(H) and *msxC *(J) resemble that of *X. helleri*. Both genes are up-regulated in the same set of fin rays compared to untreated controls (L). Expression of both genes (I, K) at 10 dt is comparable to that of *X. helleri *with species-specific differences in the shape of growing rays. Black arrowheads indicate the expression in the distal part of the fin rays, white arrowheads indicate inter-ray expression. (*X. helleri*: n = 10 for every stage and probe; *X. maculatus*: 5 dt: n = 5; 10 dt and controls: n = 3; scale bars: A, C, D, F-L: 200 μm; B and E: 100 μm).

At 5 days of testosterone treatment, strong expression of *fgfr1 *was found in the distal part of the main gonopodial rays 3, 4 and 5, the so-called 3–4–5 complex [34] of *X. helleri *(Figure 4A). Because gene expression in deeper layers of fin rays may be shielded from detection during whole mount *in situ *hybridisation [35], we performed *in situ *hybridisation on longitudinal sections which reveal strongest expression of *fgfr1 *in mesenchymal cells at the tip of growing gonopodial rays (Figure 4B). This pattern persists during later stages of gonopodium development (Figure 4C). In addition, *fgfr1 *is up-regulated in the interray tissue (Figures 4A and 4C).

As in developing swords, the spatio-temporal expression pattern of *fgfr1 *is similar to that of *msxC*, which is up-regulated in the mesenchyme of gonopodial rays 3 to 5 and in interray tissue (Figures 4D–F). Both genes are not up-regulated in untreated fins (Figure 4G).

The spatio-temporal expression pattern of *fgfr1 *and *msxC *in developing gonopodia of the platyfish *X. maculatus *approximately resembles the pattern found in *X. helleri*. Both genes are up-regulated in the distal part of the gonopodial rays 3, 4 and 5 and in the interray tissue (Figures 4H, I, J, K) compared to control fins (Figure 4L). The different shapes of the distal fin ray tips in *X. maculatus *and *X. helleri *are due to species-specific differences between the gonopodia [33].

### *fgfr1 *and *msxC *show similar expression profiles in regenerating swords

**Figure 5:**
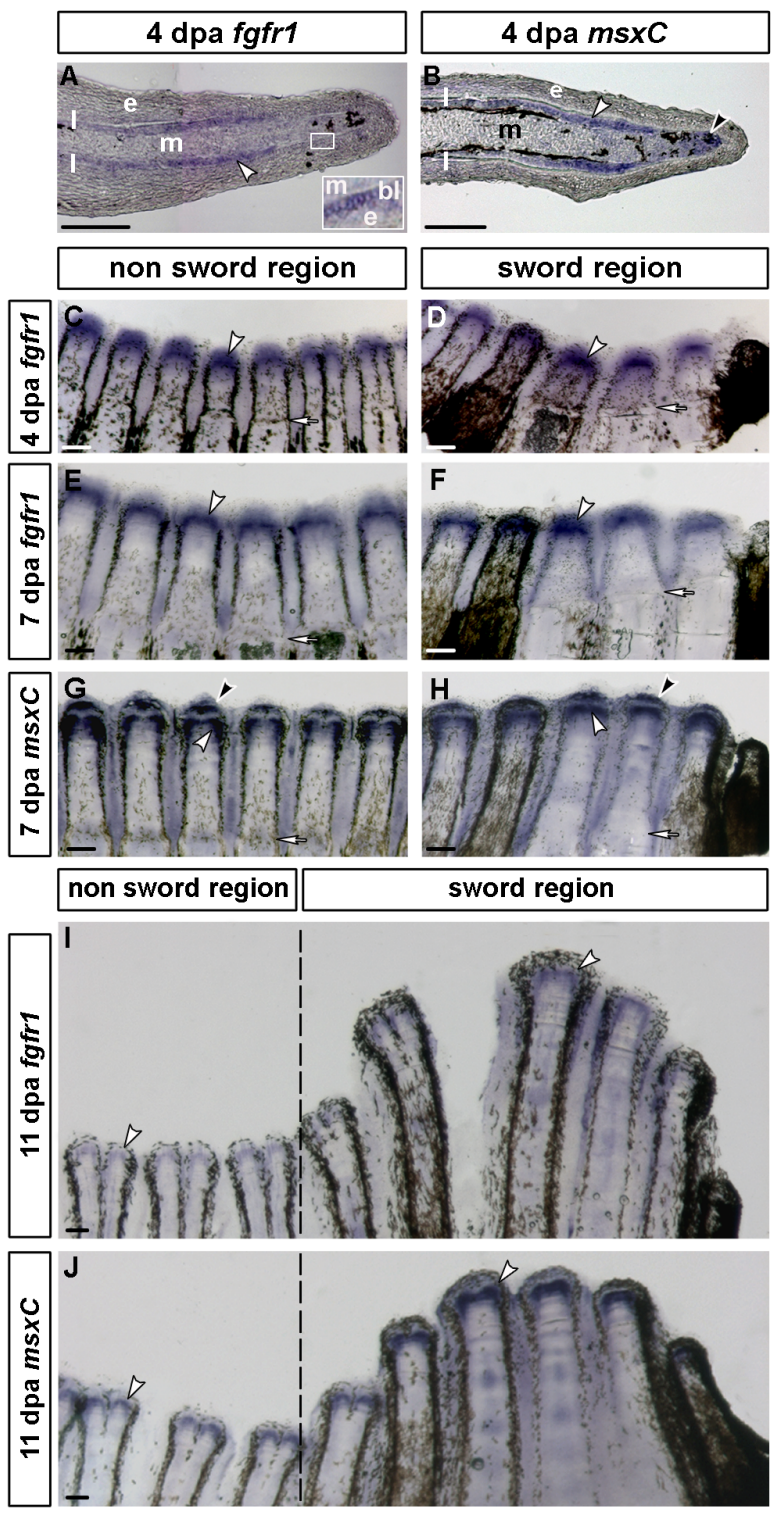
**Expression of *fgfr1 *and *msxC *during caudal fin regeneration**. *fgfr1 *is expressed in the regenerating caudal fin blastema. In situ hybridisation on longitudinal sections at 4 days post amputation (dpa) reveal *fgfr1 *expression in the basal layer of the epidermis and in scleroblasts (A). *msxC *expression overlaps with thatof *fgfr1 *in scleroblasts (B) and shows additional expression in the distal blastema (B, G, H). There is no overall clearly visible difference in expression of *fgfr1 *(C-F) and *msxC *(G, H) between sword and non-sword regenerates until 7 dpa. At 11 dpa *fgfr1 *(I) and *msxC *(J) show higher levels of expression in regenerating sword rays than in non-sword rays, though this difference is more obvious for *msxC*. White arrowheads indicate expression in scleroblasts, black arrowheads the *msxC *expression domain in the distal blastema and white arrows the plain of amputation. bl = basal epidermal layer; db = distal blastema; e = epidermis; l = lepidotrichia; m = mesenchyme (4 dpa *fgfr1*: n = 8; 7 dpa *fgfr1*: n = 5; 7 dpa *msxC*: n = 5; 11 dpa *fgfr1*: n = 5; 11 dpa *msxC*: n = 4; scale bars: A and B: 100 μm, C-J: 200 μm).

High levels of *msxC *transcription are also associated with regenerating sword rays after amputation [23]. It is assumed that the general mechanisms of growth control that act during early development are re-established during regeneration [36,37]. To test whether *fgfr1 *is similarly regulated in regenerating and in developing sword rays, we assayed gene expression in caudal fin blastemata. The regeneration kinetics of *X. helleri *roughly equals that of zebrafish at 25°C, where the regenerative outgrowth starts at ~4 dpa [38]. *fgfr1 *is expressed in the basal layer of the epidermis and in a proximal region, which are likely to be scleroblasts (Figure 5A). *msxC *and *fgfr1 *expression overlap in these cells. Furthermore, *msxC *is not expressed in the basal epidermal layer, but transcription is high in the distal blastema (Figure 5B). Sword rays and non-sword rays show similar levels of *fgfr1 *and *msxC *at different stages of regenerative outgrowth (Figures 5C–F and Figures 5G, H). Both genes stay highly up-regulated in growing blastemata until 7 dpa (Figures 5E–H).

At 11 dpa, when the sword region has begun to overgrow the rest of the regenerate, *fgfr1 *and *msxC *become differently regulated in sword rays compared to other rays. Both *fgfr1 *(Figure 5I) and *msxC *(Figure 5J) are more strongly expressed in sword rays than in non-sword rays, even though this difference in expression was more clearly observed for *msxC*. Judging from these data, it is apparent that both genes are similarly regulated in developing as well as regenerating swords. Furthermore, it is likely that due to the lack of *fgfr1 *expression in the distal blastema, *msxC *expression in this domain is regulated by factors other than Fgfr1.

### *fgf24 *and *fgf20a *are expressed in regenerating, but not developing swords

**Figure 6:**
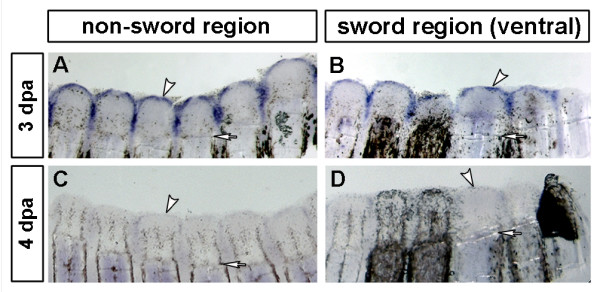
**Expression of *X. helleri fgf24 *during fin regeneration**. *fgf24 *is expressed in the wound epidermis at 3 dpa (A, B). Expression diminishes after 3 dpa and is almost absent by 4 dpa (C, D). *fgf24 *is not differentially expressed in sword rays (B) compared to non-sword rays (A). White arrowheads indicate the expression in wound epidermis and white arrows the level of amputation. (3 dpa *fgf24*: n = 6; 4 dpa *fgf24*: n = 5; scale bars: 200 μm).

To further analyse the regulation of sword development and regeneration upstream of *fgfr1*, we cloned two putative ligands of Fgfr1, *fgf24 *and *fgf20a*, which are known to be involved in fin regeneration and development [27,28,39]. To this end we examined the expression patterns of both genes in developing and regenerating swords. We detected strong expression of *fgf24 *and *fgf20a *in caudal fin regenerates up to 3 dpa and ~1 dpa, respectively, before the transcription rate of both genes decreased (Figures 6A–D and data not shown). Therefore both genes are unlikely to play a role in the regulation of Fgf-signalling or *msxC *in later stages of sword regeneration, when gene regulation becomes different between sword and non-sword rays. In addition, as neither *fgf24 *nor *fgf20a *were expressed in developing swords or gonopodia (data not shown), it is unlikely that they act as ligands for Fgfr1 during these processes.

### Expression levels of *fgfr1 *and *msxC *are correlated with growth rates of regenerating fin rays

**Figure 7:**
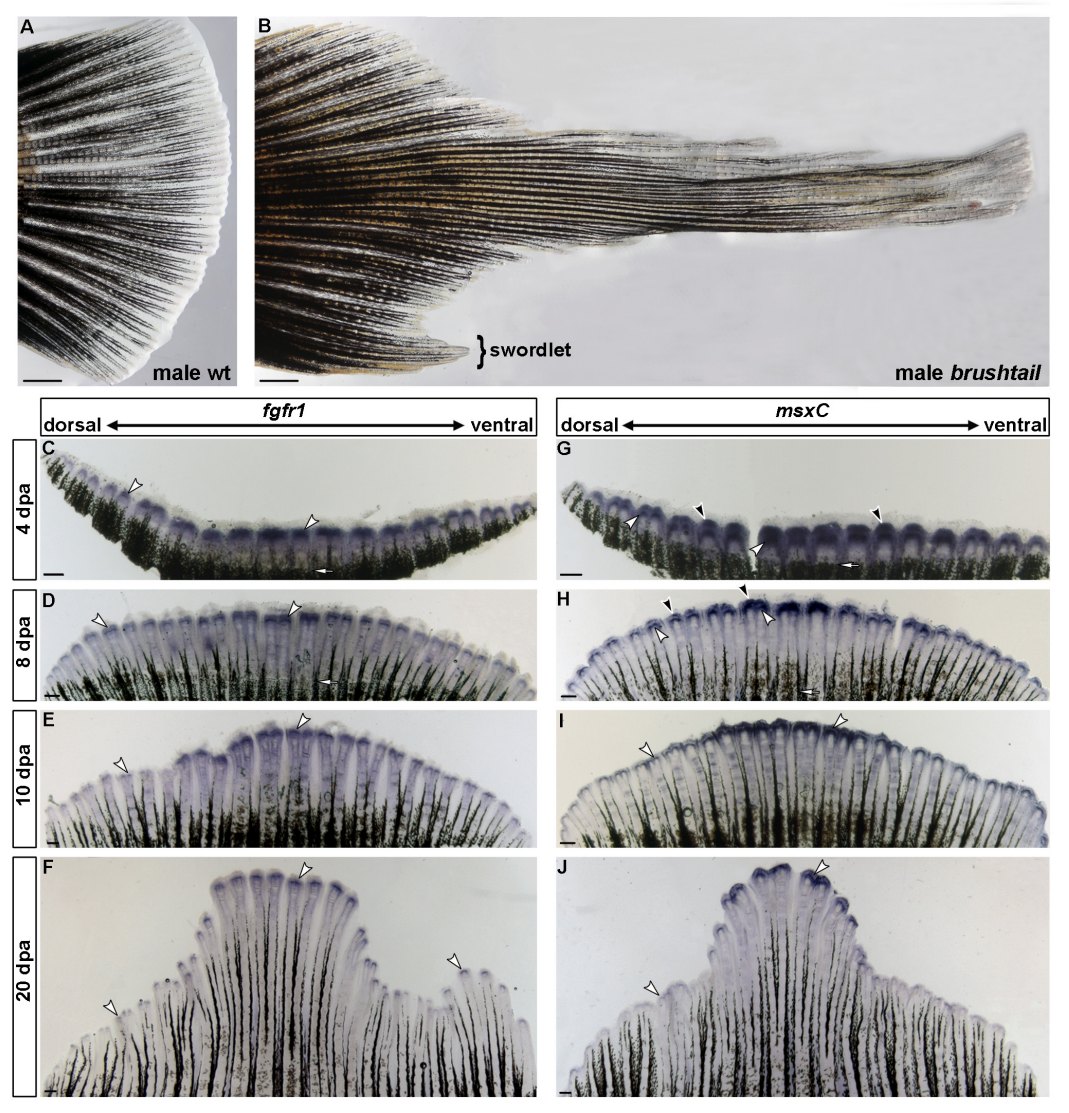
**Expression of *fgfr1 *and *msxC *in regenerating caudal fins of *brushtail *mutants**. Compared to a wildtype platyfish (A), *X. maculatus brushtail *mutants possess elongated median caudal fin rays (B). Male *brushtail *mutants also develop a small ventral extension of the caudal fin (swordlet). *fgfr1 *and *msxC *show a graded expression pattern in regenerating caudal fins of *brushtail *mutants at different stages of regeneration with strongest expression in the median fin rays (C-J). *fgfr1 *(C-F) and *msxC *(G-J) are expressed in a similar pattern as in *X. helleri *regenerating caudal fins. At later stages of regeneration, *fgfr1 *(F) and *msxC *show stronger expression in the ventral-most caudal fin rays of males compared to females (J). White arrowheads indicate expression in scleroblasts, black arrowheads the *msxC *expression domain in the distal blastema and white arrows the plain of amputation. (n = 3 for every stage and probe; scale bars: A and B: 1 mm; C-J: 200 μm).

To address the question whether enhanced *fgfr1 *and *msxC *expression are generally associated with extended growth of fin rays, we analysed gene expression in regenerating caudal fins of *X. maculatus brushtail *mutants (Figure 1D). Individuals carrying the dominant *brushtail *mutation are characterized by a life-long overgrowth of medial fin rays in the caudal fin (compare Figures 7A and 7B), which is independent of sex or sexual maturity [40]. The mutation causing this phenotype is not known. Mature male *brushtail *mutants also grow a swordlet, a small ventral fin extension (Figure 7B), similar to the ventral caudal fin extension that naturally occurs in two species of platyfish, *X. andersi *and *X. xiphidium*, and similar to that which can be artificially produced by high levels of exogenous testosterone in some species of platyfish such as *X. maculatus *[20,22]. However, it lacks the pigmentation pattern typical of swords in swordtails. Since *brushtail *mutants are already born with a brush [40] and developing embryos are not viable when extracted from their mothers, we asked whether *fgfr1 *and *msxC *are differently expressed in regenerating brush rays, compared to more dorsal or ventral caudal fin rays. Expression of *fgfr1 *and *msxC *is strongest in the median fin rays (Figures 7C–J), which becomes particularly obvious after 4 dpa. Both genes show a graded expression pattern with a decrease of expression levels towards the dorsal and ventral fin margins (Figures 7D–F, H–J). At later stages of regeneration *fgfr1 *and *msxC *are also stronger expressed in the ventral-most caudal fin rays of males that form the swordlet, but was absent in females (compare Figure 7F to 7J, and not shown).

**Figure 8:**
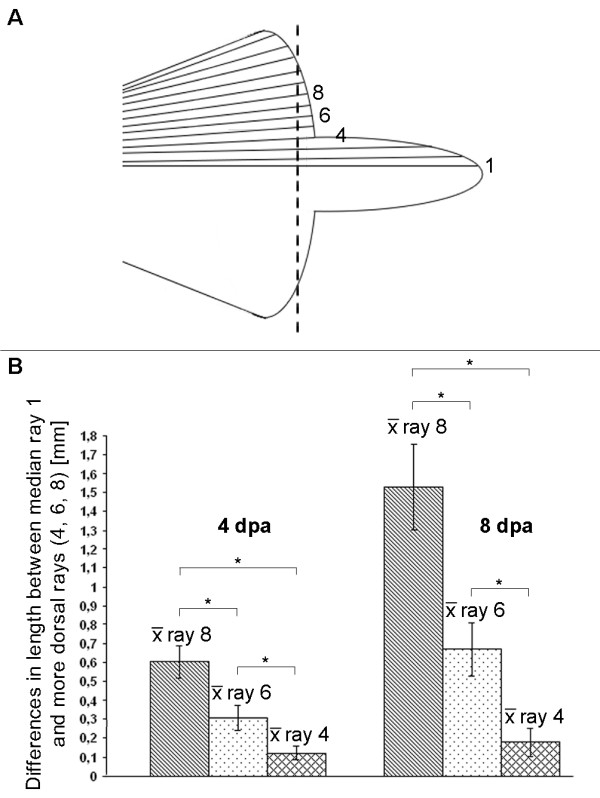
**Different regeneration rate of *brushtail *fin rays, depending on their position in the caudal fin**. The regenerate's length of four different fin rays, highlighted in the schematic drawing of an adult *brushtail *fin (A) were measured at 4 days post amputation (dpa) and 8 dpa. The regenerate's length of the dorsal fin rays 4, 6 and 8 were then compared to that of the median fin ray 1. Dorsal fin rays regenerate more slowly than the median fin ray 1, shown as average length difference between fin ray regenerates (B). The difference in regeneration rate increases the closer a fin ray is located to the dorsal edge of the fin. The position dependence of regeneration rates is more obvious at 8 dpa (n = 11; *P < 0.00001, *t*-test).

The graded expression patterns suggest that *fgfr1 *and *msxC *correlate with different growth rates of median fin rays compared to more ventral or dorsal rays. In order to test this hypothesis, we amputated the caudal fins of adult *brushtail *mutants and compared the growth rates of regenerating fin rays at different positions within the caudal fin. We did this by calculating the average length difference between the regenerate of the median fin ray 1 and more dorsal fin rays 4, 6 and 8 at 4 dpa and 8 dpa (Figure 8A). We found that the individual fin rays show significantly different regeneration rates (as determined by a *t*-test), depending on their position within the caudal fin, with the median-most ray 1 showing the fastest regeneration rate (Figure 8B). The regeneration rate decreases the more closely a fin ray is located to the dorsal edge of the fin, with regenerating ray 8 showing the slowest regeneration rate (Figure 8B). Differences in regeneration rates between fin rays according to their position in the caudal fin are more pronounced at 8 dpa (Figure 8B). The correlation between higher *fgfr1 *and *msxC *expression levels and enhanced regenerative outgrowth suggests that both genes are involved in modulating the growth rate of individual fin rays.

## Discussion

The sword is a sexually selected trait in swordtail fish that is thought to have evolved in the common ancestor of all extant *Xiphophorus *fishes, and was lost secondarily in the lineage leading to the platyfish [5,9,10]. Questions about the evolution and the subsequent loss of the sword can therefore be cogently formulated within this phylogenetic framework. Zander and Dzwillo coined the term "sword genes" for those genes that confer an ability to produce a sword in hybrids of platyfish and swordtails [18]. We employed a candidate gene approach to more broadly identify genes involved in sword development. We reasoned that genes that act in growth-dependent processes like fin regeneration might also be suitable candidates for regulating growth in growing fin rays. We therefore evaluated changes to the expression of genes involved in Fgf-signalling, a signalling pathway known to control outgrowth, and *msxC *expression in regenerating caudal fins of zebrafish [25,28,30].

### *fgfr1 *expression in developing and regenerating swords

Our results show that *fgfr1 *is specifically up-regulated in growing fin rays of the sword and the gonopodial rays of the 3–4–5 complex as a response to the induction of male sexual trait formation by exogenously supplied testosterone. Several studies suggest that Fgfr1 plays essential roles during appendage formation and regeneration: expression of *fgfr1 *is re-established during regeneration of limbs and caudal fins [25,41], and loss of *fgfr1 *function in these tissues blocks blastema formation and regenerative outgrowth [25,26,42]. Moreover, Fgfr1 is involved in supporting outgrowth of the mouse limb bud by maintaining mesenchymal cell survival and influencing the development and identity of digits [43,44]. Interestingly, the spatio-temporal expression pattern of *fgfr1 *generally overlaps with that of the transcription factor *msxC *in developing swords and gonopodia [23]. *msx *genes are known to keep cells in an undifferentiated state by promoting cell proliferation [45,46]. In zebrafish, *msxB *has been shown to regulate cell proliferation in the distal blastema of regenerating caudal fins [26,47]. Accordingly, knock-down and chemical inhibition of Fgfr1-signalling abolishes the expression of *msxB *and *msxC *[25,26]. Therefore, as judged by the up-regulation in growing sword and gonopodial rays, we propose that an increase in *fgfr1-*mediated signalling regulates cell-proliferation, similar to the processes in fin regeneration, through the activation of target genes like *msxC*.

Are there positional values that bias the ventral caudal fin for sword development? In a set of transplantation experiments it has been shown that the two major sword rays, V8 and V9, act to organize flanking rays into the developing sword [19]. This is achieved through a non-autonomous signalling process in which fin rays are induced to grow more strongly the closer they develop to V8 and V9. The development of sword rays is initiated through androgen-signalling in maturing juvenile fish, which leads to mainly autonomous growth of rays V8 and V9 and inductive signalling to flanking rays V7 and V10, which together comprise the mature sword. Moreover, the potential to develop organizing activity in V8 and V9 is established during embryonic or larval development [19]. In zebrafish ventral tail fin tissue has been fate-mapped to late gastrula stage embryos [48,49]. It is therefore likely that positional information within the *Xiphophorus *caudal fin is also attained during embryonic stages. Genes of the TGFβ gene family, such as *alk8*/*lost-a-fin *or *tolloid/mini fin*, whose mutant phenotypes include defects of the ventral caudal fin [50,51], could therefore provide all or part of the positional information that is used to specify sword ray fate.

To test whether *fgfr1 *and *msxC *are also differently regulated in regenerating swords, and to further characterize these interactions at the cellular level, we compared expression in regenerating sword and non-sword rays. An overlap of *fgfr1 *and *msxC *expression was detected in scleroblasts. In addition, *fgfr1 *is expressed in the basal layer of the epidermis, where *msxC *was not found, suggesting that signalling through *fgfr1 *alone is not sufficient to activate *msxC *expression in the basal epidermal layer. Likewise, *msxC *is temporally activated in the distal blastema, although *fgfr1 *is absent from this domain, suggesting that *msxC *is regulated by signals other than *fgfr1 *in the distal blastema. Interestingly, such a distal expression domain of *fgfr1 *is described in zebrafish [25], indicating species-specific differences in *fgfr1*- and, probably, in *msxC*-regulation between *X. helleri *and *D. rerio*. This may not be all that surprising since these two fish species are evolutionary rather distantly related [52,53]. In addition, functional differences between zebrafish and medaka *fgfr1 *have recently been demonstrated [54]. We also noted that, although expression levels of *fgfr1 *are clearly elevated in sword rays, when compared to non-sword rays, the difference was never as strongly as that observed for *msxC*. A disproportionally higher activation, like for *msxC*, might be expected in a scenario where *msxC *expression is up-regulated as part of the downstream effects of signalling through Fgfr1.

### *fgfr1 *and *msxC *are part of a network that regulates sword development

We previously proposed that the gene regulatory network underlying sword development did not evolve anew, but was co-opted from the gonopodium [23]. The 3–4–5 complex of the gonopodium is likely to have evolved only once in the common ancestors of all poeciliid fish and is thus evolutionary older than the sword, which originated in its fully developed form in the lineage leading to the genus *Xiphophorus *[5,32,34]. In support of this view, the regulatory dynamics of *fgfr1 *and *msxC *show very similar expression patterns during gonopodium growth and caudal fin regeneration that are conserved between swordtails and platyfish, whereas differences in later stages of gonopodium development are likely to reflect differences in species-specific morphology [33]. The similarities in gene expression between swords and gonopodia supports the hypothesis that sword development evolved by co-option of an androgen-regulated genetic network from the evolutionary older gonopodium. *fgfr1 *is most likely one of a number of regulatory nodes within an intricate network of signalling pathways that converge on the activation of *msx *genes. The observation that there is only partial co-expression between *fgfr1 *and *msxC *during regeneration supports this notion.

There is compelling evidence that *msx *genes are also regulated by Bmp signalling during development and regeneration of vertebrate appendages [55-57]. For example, inhibition of *BMP *signalling in the developing mouse limb leads to down-regulation of *Msx2 *[57], while over-activation of the pathway results in up-regulation of *Msx1 *and *Msx2 *[55]. Activation may be direct, as Bmp4 induces the interaction of Smads with *Msx1 *regulatory sequences [58]. Bmp-regulation of *Msx *genes seems to be widely conserved, since inhibition of Bmp signalling also leads to down-regulation of *msxb *in regenerating fins [56] and to a loss of *Msx1 *expression in the regenerating tail of *Xenopus *tadpoles [59]. Wnt signalling is a third component of this regulatory network. Manipulation of its activity in regenerating limbs, fins and tadpole tails showed that Wnt signalling regulates the expression of *msx *and *fgf *genes and is important for regenerative outgrowth [60-62]. Recent work in the poeciliid fish *Gambusia affinis *had shown that induction of *sonic hedgehog (shh) *expression by androgens is required for gonopodium formation [63]. Given that Shh controls dermal bone development in the zebrafish caudal fin [47], it is likely that also Shh signalling may be required for sword development. Functional tests will be required to dissect the molecular network that controls *msxC *expression to better understand how these different signalling pathways act together to shape the male sword. The similarities in gene expression between swords and gonopodia supports the hypothesis that sword development evolved by co-option of a modular androgen-regulated *fgfr1/msxC *network from the evolutionary older gonopodium.

**Figure 9:**
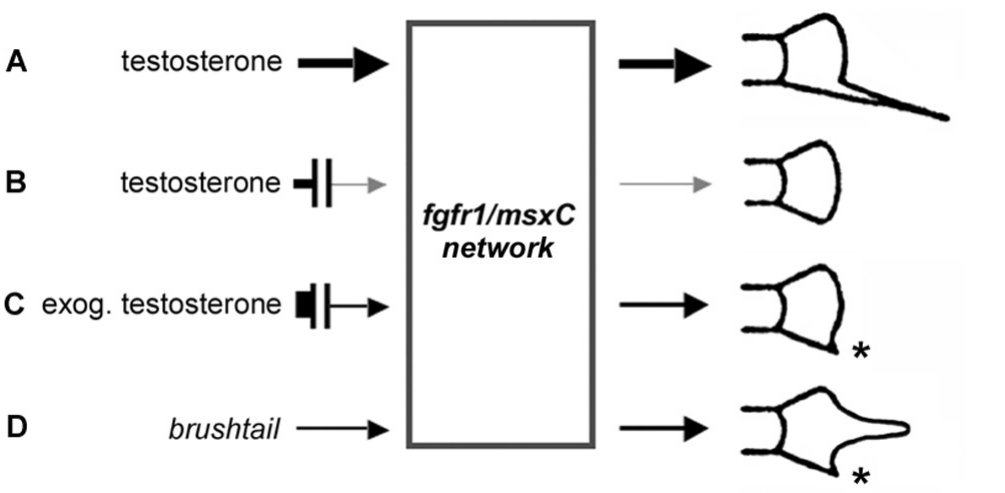
**Growth control in prospective sword rays in the ventral caudal fin**. An signalling network that includes activation of *fgfr1 *and *msxC *expression regulates growth of ventral rays in the caudal fin and is activated by endogenous levels of testosterone in swordtails (A); in platyfish an evolutionary weakening (interrupted arrow) in the ability of testosterone to activate the network results in insufficient signalling and the absence of sword development (B); high exogenous levels of testosterone overcome the inhibition upstream of the network and induce a swordlet in some platyfish species (C); the platyfish mutant *brushtail *raises overall signalling levels of the network in all rays, allowing endogenous testosterone to suffice for the induction of a swordlet in male fish (D). Width and shading of arrows indicate the strength of *fgfr1/msxC *signalling activation in sword rays (wide black: induction of a sword; narrow grey: maintenance of basal fin growth; narrow black: induction of a swordlet).

Juveniles of *X. maculatus *fail to up-regulate *fgfr1 *and *msxC *in the ventral caudal fin rays at testosterone-levels that otherwise cause sword development in swordtails after 4 days of treatment. It is only after prolonged exposure that both genes start to be expressed more strongly in the ventral-most rays. This expression marks the development of a "swordlet", a small colourless caudal fin extension, that develops in some platy species under excessive exposure to testosterone [20,22]. A swordlet is also found naturally in the species *X. andersi *and *X. xiphidium *and outside the genus *Xiphophorus *in other species of poeciliid fishes, e.g. in *Poecilia petenensis*. The failure to up-regulate the *fgfr1/msxC *network in response to endogenous androgens may have caused the loss of swords in the lineage leading to the platyfish. Since artificially high levels of testosterone can overcome this inhibition ([20,22] and Figure 3), the evolutionary changes that led to sword-loss may have been quantitative ones that could have altered the strength of genetic interactions within the *fgfr1/msxC *network or its upstream regulation. One attractive possibility for the evolutionary loss of swords therefore would be an acquired loss in the sensitivity of the *fgfr1*/*msxC *network to be activated by androgen-signalling (Figure 9), possibly at the level of *fgf *regulation.

### *brushtail *enhances sword development in platyfish

We analysed fin ray regeneration in *X. maculatus brushtail*, a mutant that spontaneously arose in platy strains within the pet trade (Figure 1D). The brush resembles the sword with regard to size, but occurs in both sexes, consists of medial fin rays, and lacks conspicuous pigmentation [40]. In contrast to swords, brushes are formed during embryonic or larval development and show allometric growth throughout the whole life of an individual. In adult male platyfish, *brushtail *often also induces a swordlet on the ventral caudal fin margin (M. Schartl, personal communication). The *X. maculatus brushtail *fish used in our studies are not congenic and may contain alleles derived from hybrids between swordtails and *X. maculatus*. It could thus be argued that *brushtail *hybrids are more inclined to develop swords due to introgression of sword alleles. However, we note that formation of the swordlet in males is always linked to the *brushtail *phenotype and does not occur in male brushless siblings that derive from a cross between *brushtail *parents. Therefore *brushtail *enhances a dormant program of sword development [64], similar to prolonged exposure to testosterone in *X. maculatus*. We thus reasoned that the mechanisms of fin growth in swords and brushes may be similar. Our analysis of fin ray regeneration revealed that (1) regeneration rates in *brushtail *and swordtail caudal fins differed in that sword rays regenerate at the same rate as the rest of the fin (Figures 5 and 7 and [23]), whereas rays within the brush exhibit accelerated regeneration rates the closer they are to the middle of the fin, and (2) expression levels of *fgfr1 *and *msxC *show a positive correlation with regeneration rates. Therefore, *brushtail *mutants lend support to the hypothesis that the strength of *fgfr1/msxC *expression is correlated with the rate of regenerative growth, which in *brushtail *mirrors the life-long overgrowth of the brush. In contrast, sword rays initially exhibit regeneration rates and strong *fgfr1/msxC *expression indistinguishable from non-sword rays. Growth and gene expression ceases once rays have reconstituted the previous fin length, while *fgfr1*/*msxC *transcription remains high in the sword. The correspondence of gene expression levels in swords, gonopodia, and in *brushtail *suggests that *fgfr1 *and *msxC *expression are a general readout of proliferation in fin rays.

Pharmacological interference with *fgfr1 *signalling is not practicable in *Xiphophorus *fish, as treatments using the standard zebrafish assay, where many fish are treated in small volumes of water, cause a high mortality, while treatments in volumes used in our testosterone experiments would require excessive amounts of the drug. Moreover, electroporation of morpholinos has failed due to persistent problems with the initial injection, which has to be performed into growing fin rays, rather than into a regeneration blastema (not shown). Therefore we had to resolve to associative studies of gene expression in a phylogenetic framework and in mutants to study the involvement of candidate genes in sword development.

As an explanation of the development of a swordlet in adult male *brushtail *platyfish, our findings suggest that *fgfr1/msxC*-mediated signalling is constitutively activated throughout the *brushtail *caudal fin and already in young larvae of both sexes, with strong activation in medial rays and lower, yet elevated levels at the margins. Although endogenous levels of testosterone in platyfish are usually not sufficient to induce or maintain a sword, growth of a swordlet could nevertheless ensue if in maturing male platyfish both testosterone- and *brushtail*-activated signalling pathways converged on the activation of the *fgfr1/msxC *regulatory network. *brushtail *simulates the effect of high exogenous doses of testosterone on swordlet formation in platyfish, in agreement with the idea that the *fgfr1/msxC *network is a major downstream target of androgen signalling during sword development (Figure 9). It is interesting to note that *brushtail *re-awakens a dormant program for sword development that had been evolutionary lost in platyfish. Therefore, swordlets likely employ at least parts of the developmental program controlling sword growth [64]. An examination of the molecular mechanisms that activate *fgfr1/msxC *signalling in *brushtail *will be valuable for exploring the regulation of this gene network in controlling the growth of ventral caudal fin rays.

### The ligands required for Fgfr1 activation during fin development remain elusive

To examine the regulation of fin growth upstream of *fgfr1*, we isolated *fgf20a *and *fgf24 *as potential ligands in caudal fins that develop a sword under testosterone treatment. In zebrafish, *fgf20a *plays an essential role in blastema formation, while *fgf24 *is expressed in regenerating caudal fins and is required for pectoral fin bud initiation [25,27,28,39]. Therefore, both genes are putative candidates to control early phases of sword and gonopodium development such as the re-initiation of fin growth under testosterone. We show that both genes are transiently expressed in regenerating fin rays and are down-regulated after 3 dpa, suggesting that *fgf20a *and *fgf24 *fulfill similar functions in blastema formation in zebrafish and *X. helleri*. Since neither gene was expressed at any detectable level in developing swords or gonopodia, other *fgfs *remain attractive candidates that might mediate the activation of sword development downstream of testosterone-activated androgen receptors. A number of other Fgfs are known with confirmed roles during appendage formation and regeneration, such as Fgf2, Fgf4, Fgf8 and Fgf10, which are able to induce regeneration in *Xenopus *limbs or chick limb buds [65-67], and Fgf16 as well as Fgf8, Fgf4 and Fgf10, which are involved in zebrafish pectoral fin development (reviewed in [68]). Further investigation of these candidate genes should uncover the ligand of Fgfr1 that controls development of the sword and gonopodium.

## Conclusion

We have shown a correspondence of *fgfr1 *and *msxC *expression levels in developing and regenerating swords, gonopodia, and in the fin-overgrowth mutant *brushtail*, which suggests that *fgfr1 *and *msxC *are part of a genetic network that regulates the rates of growth and regeneration in male-specific modifications of adult fins in swordtails and platyfish. Through inter-species comparisons with swordless platyfish, we have shown that high levels of *fgfr1 *expression are associated with developing sword rays. In line with this assertion *brushtail *mutant caudal fin rays exhibit elevated expression of this network, leading both to a regionalised fin overgrowth phenotype and to an enhancement of an otherwise dormant program of sword development in platyfish. Taken together, we propose that changes in the regulation of a genetic network that includes *fgfr1 *and *msxC *contribute to the loss of the sword in the platyfish lineage. Our characterization of the two known regulators of Fgf signalling in teleost fin regeneration, *fgf20a *and *fgf24*, rules out an involvement in sword development, but other Fgfs remain candidates that might mediate the activation of sword development downstream of testosterone-activated androgen receptors. Finally, the similarities in gene expression between swords and gonopodia supports the hypothesis that sword development evolved by co-option of an androgen-regulated *fgfr1/msxC *network from the evolutionary older gonopodium.

## Methods

### Fish stocks and maintenance

*Xiphophorus helleri *and *X. maculatus *were taken from stocks kept at the animal research facility at the University of Konstanz. The *X. maculatus brushtail *mutant, a commercial breed, was obtained from a local pet shop. Fish were maintained on a 12:12 h light:dark cycle at 24°C in 110-litre densely planted aquaria and were fed TetraMin flakes and Artemia.

### Testosterone treatment and fin regeneration

Up to six juvenile individuals of *X. helleri*, aged between 3 and 6 months, were placed in a 30-litre tank. 17-α-Methyltestosterone (1 mg/ml stock solution in ethanol; Sigma-Aldrich, Munich, Germany) was added to the water twice a week to a final concentration of 10 μg/l. After 5 or 10 days of testosterone treatment approximately 1/3 of the distal part of the caudal fin and approximately 2/3 of the anal fin was amputated using a sterile razor blade. For fin amputation fish were anesthetized by incubation in a solution of 0.08 mg/ml tricaine (3-aminobenzoicacid-ethylester-methanesulfonate; Sigma-Aldrich, Munich, Germany).

For regeneration experiments adult *X. helleri *and *X. maculatus *individuals were anesthetized and 1/3 of the caudal fin was amputated. Subsequently, fins were allowed to regenerate at 24°C for variable time periods, without addition of 17-α-Methyltestosterone, depending on the experiment. Fish were anesthetized again and the blastema was removed. Fins and blastemata used for *in situ *hybridisation were fixed in 4% paraformaldehyde (Sigma-Aldrich, Munich, Germany) in phosphate buffered saline (PBS) overnight, transferred to methanol and stored at -20°C until use.

### Cloning *fgfr1*, *fgf24 *and *fgf20a*

Total RNA was isolated from caudal fin blastemata 24 hpa (for *fgf20a*), 48 hpa (for *fgf24*) and 72 hpa (for *fgfr1*) and used for cDNA synthesis as described [23]. Degenerate Primers were designed based on an alignment of cDNAs from *Danio rerio*, *Tetraodon nigroviridis *and *Takifugu rubripes *to amplify cDNA fragments of the desired genes. A 1299 kb *fgfr1 *fragment was amplified by PCR using the Primer pair fgfr-fw1: 5'-CCIGAIAARATGGARAARAARYTGCAYGC-3' and fgfr-rev1: 5'-CIGGIACYTGGTMIGGRTTRTARCA-3'. A 605 bp *fgf24 *fragment was amplified by PCR using the Primers fgf24-fw1: 5'-GAKAGIGCARCRIGYIYGGAIRC-3' fgf24-rev1: 5'-GTCCICYYIKCCYTTKGGYTGGCGC-3' and fgf24-rev2: 5'-CCAGTATAAATAAMAYRACAGACAC-3'. A 497 bp *fgf20a *fragment was amplified by PCR using the Primer pair fgf20a-fw2: 5'-GGSTCTCATTTCGTYCTCAC-3' and fgf20-rev0: 5'-GTRTTRTACCARTTYTCYTC-3'.

To obtain *fgf24*/*fgf20a *fragments of appropriate size for RNA probe generation 3'RACE reactions were performed using a 3'RACE kit (Roche). A ~1,3 kb *fgf24 *fragment was amplified using the gene-specific primers fgf24R-fw1: 5'-CTACAGCAGGACCACGGGCAAAC-3' and fgf24R-fw2: 5'-CAAGAAAGGCTCACGCACCACGC-3'. A ~1,2 kb *fgf20a *fragment was amplified using the gene-specific primers fgf20a-fw1: 5'-CATCAGAGGAGTGGACAGCGGC-3' and fgf20a-fw2: 5'-CGCCATGAACAGCAAGGGGGAG-3'. The PCR products were gel-purified using the QIAquick Gel Extraction Kit (Qiagen, Hilden, Germany) and cloned into the pCRII-TOPO vector (Invitrogen, Karlsruhe, Germany) for sequencing.

### Whole-mount in situ hybridisation

Antisense and sense RNA probes were generated using a digoxigenin labelling kit (Roche, Mannheim, Germany). Probes for *fgfr1, fgf24 *and *fgf20a *were generated from the cDNA fragments listed above. *msxC *probes were generated from a 635 bp cDNA fragment [23]. Because of the high sequence conservation within the genus, *X. helleri *antisense probes could also be used in the platyfish *X. maculatus*.

In situ hybridisation of *Xiphophorus *fins and blastemata were performed as described [25] with several modifications. Prehybridisation was done 4 h at 68°C in formamide solution (50% formamide, 5× SSC, 0,1% Tween-20, pH to 6 with 1 M citric acid). Post-hybridisation washing steps were initiated at 68°C with formamide solution. To block unspecific binding sites 0,5% blocking reagent (Roche, Mannheim, Germany) in PBST (PBS/0.1% Tween-20) was used. Antibody incubation was done at 4°C overnight. After fixation of stained fins/blastemata, the tissue was washed twice for 20 min in PBST, 20 min in ethanol/PBST (70:30) and 20 min in 100% ethanol and stored at 4°C. The specificity anti-sense probes were verified with sense probe experiments.

### *In situ *hybridisation on longitudinal sections

4 day-old caudal fin blastemata and anal fins from individuals treated with 17-α-Methyltestosterone for 5 days were fixed in 4 % Paraformaldehyde (Sigma-Aldrich, Munich, Germany). Longitudinal sections of 10 μm thickness were created using a Reichert-Jung Autocut 2040 Microtome and *in situ *hybridisation was performed as described [69].

### Microscopy and image editing

Fin explants and *brushtail *caudal fins were analysed using a Zeiss Stemi SV11 Apo. Pictures were taken using the AxioVision software v3.1 (Zeiss, Jena, Germany) and the digital camera Zeiss AxioCam MRc. The pictures were processed using Adobe Photoshop.

### Fin ray measurement and calculation

Pictures from regenerating caudal fins of males and females were photographed at 4 and 8 days post amputation. The regenerate length of the median fin ray 1 and more dorsal fin rays 4, 6 and 8 was measured using the software ImageJ [70]. In case of bifurcation both semi-rays' regenerates were measured and the average was calculated. To eliminate variation in regeneration speed between the individuals, the difference in length between the regenerate of the median ray 1 and the three other ray regenerates were calculated for each fish. Last, the average was calculated for each difference and graphically presented using Microsoft Excel. A paired, one-tail *t*-test was used to determine whether the calculated average distances differ significantly from each other.

### Phylogenetic analysis and motif prediction

Phylogenetic trees of *fgf receptors*, the *fgf8/17/18/24 *and the *fgf9/16/20 *subfamily were constructed using Maximum likelihood (ML) and Bayesian methods of phylogeny inference [71]. ML analyses were performed using PHYML [72]. The best fitting models of sequence evolution for ML were obtained by ModelTest 3.7 [73]. ML tree topologies were evaluated by a bootstrap analysis with 500 replicates [74]. To confirm obtained Tree topologies Bayesian analyses were initiated with random seed trees and were run for 100,000 generations for *fgf receptors *and *fgf8/17/18/24 *and 1000,000 generations for *fgf9*/*16*/*20*. The Markov chains were sampled at intervals of 100 generations with a burn in of 100 trees for *fgf receptors *and *fgf8/17/18/24 *and 500 trees for fgf9/16/20. Bayesian phylogenetic analyses were conducted with MrBayes 3.0b4 [75] using the general time reversible model GTR+I+G [76] for *fgf receptors *and *fgf8/17/18/24 *and the Tamura-Nei model TrN+I+G [77] for *fgf9/16/20*. The sequences used for the analysis are listed in Table 1. Sequences that could not be aligned with confidence were excluded from the analysis. ScanProsite [78] was used to predict conserved motifs in the translated amino acid sequences.

**Table 1:** Sequences used for the phylogenetic reconstruction

**cDNA**	***Danio rerio***	***Tetraodon nigroviridis***	***Takifugu rubripes***	***Homo sapiens***
***fgfr1***	AF389400	GSTENT00034863001	SINFRUT00000128473	NM_000604
***fgfr2***	NM_178303	GSTENT00025098001		NM_000141
***fgfr3***	NM_131606	GSTENT00023332001	SINFRUT00000174828	M58051
***fgfr4***	NM_131430	GSTENT00014622001	SINFRUT00000143771	NM_213647
***fgf8***	NM_131281	scaf13770 (47766 to 43187)	scaffold_52 (147851 to 151899)	AF520763
***fgf17b***	BC083269	SCAF7880 (181 to 1923)	scaffold_4659 (4421 to 2507)	
***fgf17***				AY358869
***fgf18***	NM_001013264	SCAF7783 (655 to 6588)	scaffold_362 (111038 to 104939)	AY358811
***fgf24***	NM_182871	SCAF14744 (1671104 to 1664185)	scaffold_70 (668977 to 661812)	
				
***fgf10***	BC094986			
***fgf20a***	NM_001037103	GSTENT00035824001	SINFRUT00000135793	
***fgf20b***	NM_001039172	GSTENT00005561001	SINFRUT00000137831	
***fgf20***				NM_019851
***fgf16***	NM_001040407	GSTENT00017034001	SINFRUT00000152407	NM_003868
***fgf9***				BC103979

	***Mus musculus***	***Gallus gallus***	***Rattus norvegicus***	***Ciona intestinalis***

***fgfr1***	BC033447	NM_205510		
***fgfr2***	NM_010207	NM_205319		
***fgfr3***	NM_008010	NM_205509		
***fgfr4***	BC033313			
***fgfr5***	AF321300			
***fgf8***	BC048734	NM_001012767		
***fgf17***	NM_008004			
***fgf18***	NM_008005	NM_204714		
***fgf20***	BC125509	ENSGALT00000022223		
***fgf16***	AB049219	NM_001044650		
***fgf9***		AB108842	NM_012952	
***fgf9/16/20***				NM_001032477

## Authors' contributions

NO, AM and GB conceived this study, NO and GB evaluated the data. NO performed most of the laboratory work. NB contributed to the analysis of the *X. maculatus brushtail *mutant. NO, GB and AM wrote the manuscript, which was read and revised by all authors.

## Supplementary Material

Additional file 1 **Sequence and domain structure of *X. helleri fgfr1***. The 1248 bp *fgfr1 *sequence from *X. helleri *(A) codes for parts of the IG domain 2 (blue), IG domain 3 (blue) and parts of the tyrosine receptor kinase (red). B shows a schematic drawing of the isolated cDNA fragment and the domain-coding portions. Click here for file

Additional file 2 **Sequence and domain structure of *X. helleri fgf24 *and *fgf20a***. The 633 bp *fgf24 *sequence from *X. helleri *represents the full ORF of the gene, while the 663 bp sequence of *X. helleri fgf20a *misses a part of the 5' region of the ORF (A). The Heparin-binding growth factors/fibroblast growth factor (HBGF/FGF) family signature is marked in blue. B shows a schematic drawing of the two isolated cDNA fragments and their HBGF/FGF-coding portions. Click here for file
