# Supplementary figures and images for: Fgfr1 signalling in the development of a sexually selected trait in vertebrates, the sword of swordtail fish
Source: BMC Dev Biol. 2008 Oct 9;8:98. doi: 10.1186/1471-213X-8-98 (PMC2577654; doi:10.1186/1471-213X-8-98)

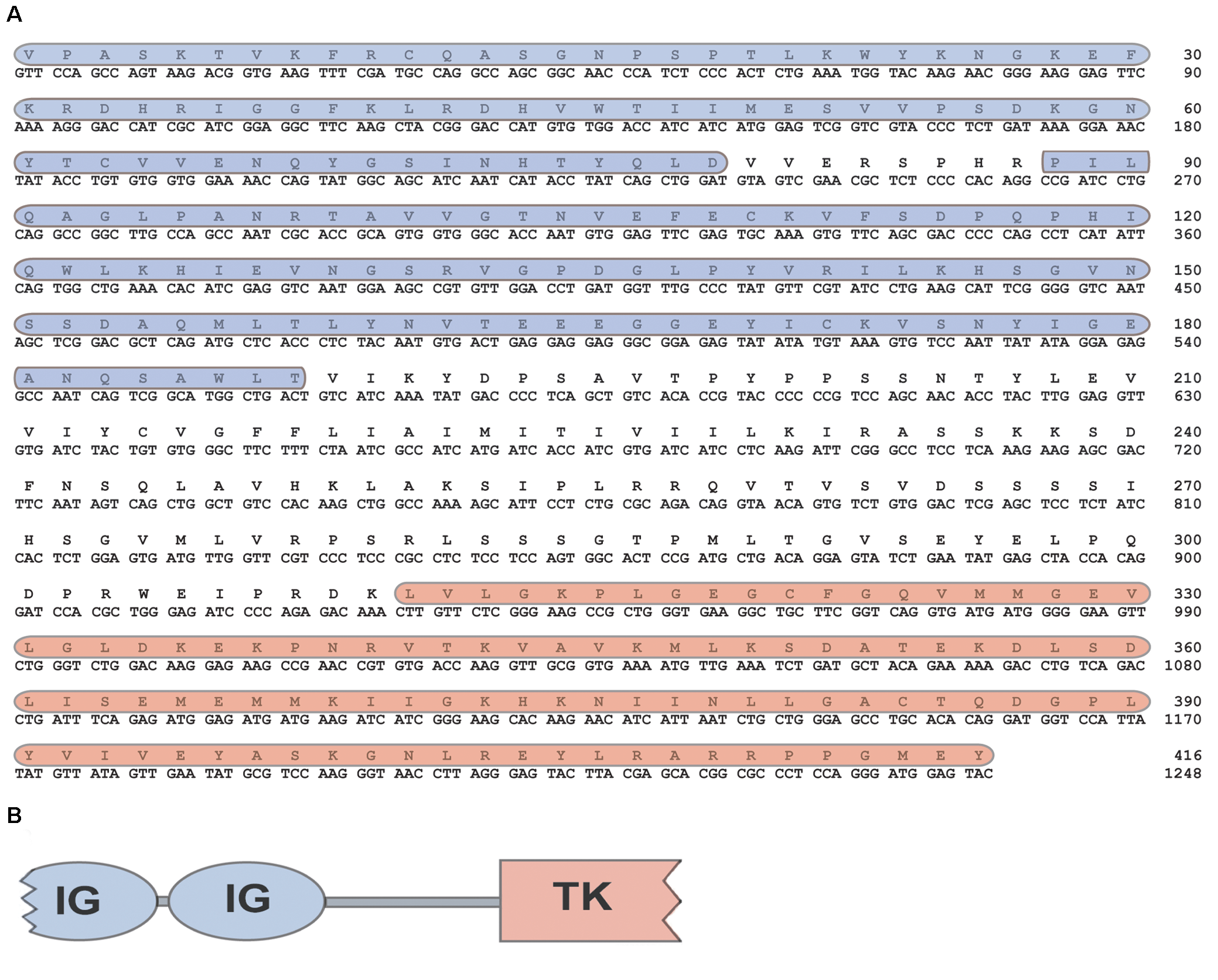

Supplement: Additional file 1 — Sequence and domain structure of X. helleri fgfr1. The 1248 bp fgfr1 sequence from X. helleri (A) codes for parts of the IG domain 2 (blue), IG domain 3 (blue) and parts of the tyrosine receptor kinase (red). B shows a schematic drawing of the isolated cDNA fragment and the domain-coding portions. [file 1471-213X-8-98-S1.png]

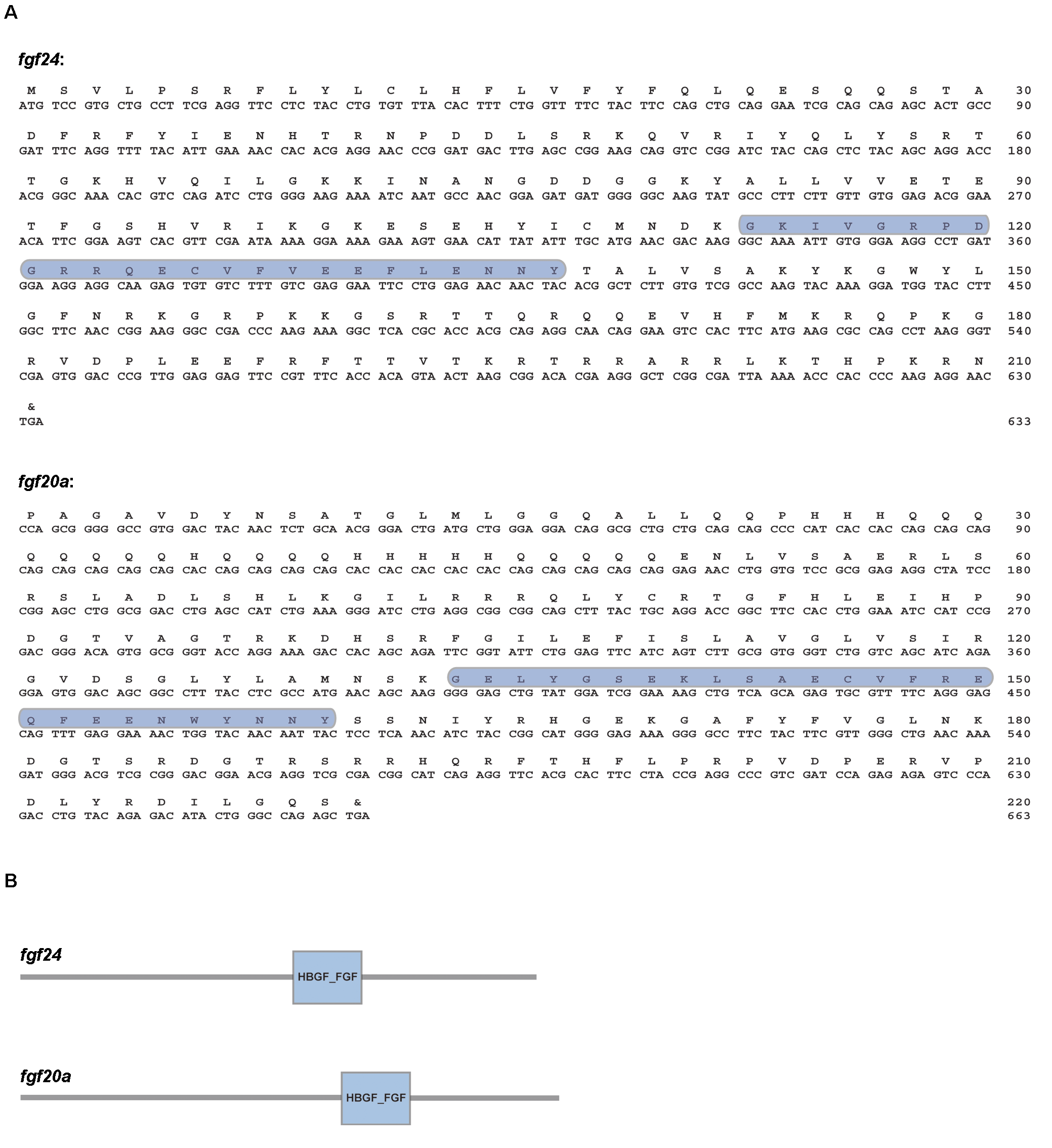

Supplement: Additional file 2 — Sequence and domain structure of X. helleri fgf24 and fgf20a. The 633 bp fgf24 sequence from X. helleri represents the full ORF of the gene, while the 663 bp sequence of X. helleri fgf20a misses a part of the 5' region of the ORF (A). The Heparin-binding growth factors/fibroblast growth factor (HBGF/FGF) family signature is marked in blue. B shows a schematic drawing of the two isolated cDNA fragments and their HBGF/FGF-coding portions. [file 1471-213X-8-98-S2.png]
